# A Positive Feedback Loop of Long Noncoding RNA LINC00152 and KLF5 Facilitates Breast Cancer Growth

**DOI:** 10.3389/fonc.2021.619915

**Published:** 2021-03-26

**Authors:** Qiang Li, Xiao Wang, Liheng Zhou, Mingyun Jiang, Guansheng Zhong, Shuguang Xu, Minjun Zhang, Yigan Zhang, Xiaodong Liang, Lei Zhang, Jianming Tang, Haibo Zhang

**Affiliations:** ^1^ Department of Radiation Oncology, Zhejiang Provincial People’s Hospital, People’s Hospital of Hangzhou Medical College, Hangzhou, China; ^2^ Department of Medical Oncology, Zhejiang Provincial People’s Hospital, People’s Hospital of Hangzhou Medical College, Hangzhou, China; ^3^ Department of Breast Surgery, Renji Hospital, School of Medicine, Shanghai Jiao Tong University, Shanghai, China; ^4^ Graduate Department, Bengbu Medical College, Bengbu, China; ^5^ Department of Breast Surgery, The First Affiliated Hospital, College of Medicine, Zhejiang University, Hangzhou, China; ^6^ The First School of Clinical Medicine, Lanzhou University, Lanzhou, China; ^7^ Department of Radiation Oncology, Renji Hospital, School of Medicine, Shanghai Jiao Tong University, Shanghai, China; ^8^ Institute of Cancer Neuroscience, Medical Frontier Innovation Research Center, The First Hospital of Lanzhou University, The First Clinical Medical College of Lanzhou University, Lanzhou, China

**Keywords:** lncRNA LINC00152, KLF5, breast cancer, positive feedback loop, cell proliferation

## Abstract

The long noncoding RNA (lncRNA) LINC00152, also known as CYTOR, displays aberrant expression in various cancers. However, its clinical value and functional mechanisms in breast cancer remain insufficiently understood. Our study found that LINC00152 is significantly upregulated in breast cancer, and that it acts as an indicator of poor survival prognosis. Further studies revealed that LINC00152 knockdown suppresses cell proliferation and tumorigenicity *in vitro* and *in vivo*. Mechanistic analyses demonstrated that LINC00152 directly binds to KLF5 protein and increases KLF5 stability. Moreover, LINC00152 is also a KLF5-responsive lncRNA, and KLF5 activates LINC00152 transcription by directly binding to its promoter. Our study suggests that LINC00152 promotes tumor progression by interacting with KLF5. LINC00152 may be a valuable prognostic predictor for breast cancer, and the positive feedback loop of LINC00152-KLF5 could be a therapeutic target in pharmacological strategies.

## Introduction

Breast cancer accounts for the most common type of malignant tumor in women (1, 2). Albeit the great advance in molecularly targeted approaches in breast cancer treatment, the desired improvement in the long-term survival was still unsatisfactory. Therefore, a potential biomarker needs to be urgently identified and its predictive molecular mechanisms in breast cancer understood. The Long noncoding RNAs (lncRNAs) was a RNA gene products which consist of 200 to 100,000 nucleotides. These transcripts have been recently identified to be largely functional but mechanistically unexplored, especially in human cancers (3). Recent evidence reveals that lncRNAs are involved in breast cancer tumorigenicity (4–6). Nevertheless, the precise mechanism by which lncRNAs regulate breast cancer tumorigenicity remains largely unknown.

The lncRNA LINC00152, also known as CYTOR, which is encoded on human chromosome locus 2p11.2, is upregulated in cancer cells and facilitate cell proliferation as well as epithelial-mesenchymal transition. It is also related to various cancers, including breast cancer (4), colorectal cancer (7), glioblastoma (8, 9), and hepatocellular carcinoma (10). It was first reported in a study of hepatocarcinogenesis (11), and its role as a crucial oncogene regulating gene expression has been observed in many types of cancers (12, 13). Recently, LINC00152 has been shown to promote cell progression through the miR-193a/b-3p/CCND1 axis in hepatocellular carcinoma (10). Other studies also indicated that LINC00152 regulates the degradation of PTEN protein *via* NEDD4-1-mediated ubiquitination and also functions as a direct target of YY1 in breast cancer (5). Interestingly, LINC00152 was reported to directly bind to Bcl-2 protein and thus activate cell cycle signaling in gastric cancer (14). Moreover, the overexpression of LINC00152 served as an independent risk factor for clinical outcome of human esophageal squamous cell carcinoma patients (15). Nevertheless, on account of the molecular and phenotypic heterogeneity, the precise biological behavior of LINC00152 in breast cancer need to be further determined.

Our study found that LINC00152 was significantly overexpressed in breast cancer and was associated with bad survival prognosis for breast cancer patients. Furthermore, LINC00152 induced breast cancer cell proliferation and tumorigenicity *in vitro* and *in vivo*. It could directly bind to KLF5 and enhances its stability. Furthermore, it was a KLF5-responsive lncRNA that serve its oncogenic function *via* KLF5-mediated manner. In conclusion, our findings indicate that LINC00152 exert as a promising prognostic predictor for breast cancer. The LINC00152-KLF5 feedback loop facilitates breast cancer progression and may serve as a potential therapeutic target in breast cancer.

## Materials and Methods

### Cell Lines

The normal breast epithelium cell line (MCF10A) and human breast cancer cell lines (BT549, MCF7, MDA-MB-468, and MDA-MB-231) were purchased from the Chinese Academy of Sciences Committee on Type Culture Collection Cell Bank (Shanghai, China). All cell lines were authenticated by Short Tandem Repeat DNA fingerprinting at Shanghai Biowing Applied Biotechnology Co., Ltd. (Shanghai, China).

### Plasmids

KLF5 and LINC00152 cDNAs from normal breast tissues were amplified by PCR. After sequencing, these cDNAs were subcloned into pcDNA3.1 vector or lentivirus pLVX-Puro vector (Clontech). After that, the promoter of LINC00152 was subcloned into pGL3 vector (Promega). shRNAs were designed to target LINC00152 site 1 (shLINC00152-1 target sequence: 5′-GCCATCATGATGGTACTTTAA-3′), LINC00152 site 2 (shLINC00152-2 target sequence: 5′-GCCAGGACACTGAGATTTGGA-3′), and KLF5 (shKLF5 target sequence: 5’-GGTTACCTTACAGTATCAACA-3’). Based on the protocol provided by the manufacturer, a QuikChange Site-Directed Mutagenesis Kit (Stratagene) was used to construct the LINC00152 point mutations.

### Tissue Samples and Clinical Data Collection

We obtained eight pairs of freshly frozen breast tumors and adjacent non-tumor specimens from Zhejiang Provincial People’s Hospital. We also collected an additional 70 pairs of formalin-fixed paraffin-embedded breast tumor and adjacent non-tumor specimens from Zhejiang Provincial People’s Hospital. None of the samples collected were from patients undergoing chemo or radiotherapy at the time of biopsy. We obtained written informed consent from the patients before commencing our analysis of the samples. The baseline information of the 70 breast cancer patients are listed in **Supplementary Excel 1**.

### Cell Proliferation and Colony Formation

Cells were seeded into the 96-well plate and incubated at the right circumstances. The WST-1 Assay Kit (Roche) was utilized to test the cell proliferation. For the colony formation assay, cells were seeded into the 12-well plate with a 0.4% top agar layer and a 0.8% bottom agar layer in triplicate. After 2–3 weeks incubation, colonies were fixed and stained by using 1% crystal violet solution, and were enumerated subsequently for analysis.

### RNA Preparation and Quantitative Real-Time PCR (qRT-PCR)

RNA preparation and qRT-PCR were performed as previously described in a study (16). In brief, TRIzol reagent (Invitrogen) was used to extract total RNA, and qRT-PCR were performed based on the manufacturer’s instructions (Thermo Fisher). The primers used in this study are shown in **Supplementary Table 1**.

### shRNA Knockdown and Transfection

The assays of shRNA knockdown and transfection were conducted as previously described in a study (17). shRNA sequences against LINC00152 and KLF5 were obtained from GeneChem (Shanghai, China). Those sequences as well as packaging plasmids were transfected into HEK293 cells. After incubation for 48 to 72 h, the cell supernatant was collected and filtered through a 0.22 μm membrane (Millipore). Then, the lentiviruses expressing shRNAs or shGFP control (8 μg/ml polybrene) were used to infect breast cancer cells. The infected cells were selected through puromycin (5 μg/ml). Multiple monoclonal cultures were screened for shRNAs by means of RT-PCR and western blotting.

### *In Situ* Hybridization

By using the RNAscope 2.5 HD Detection Reagent-BROWN kit (ACDBio), *in situ* analysis of LINC00152 interactions was conducted on paraffin-embedded sections according to the manufacturer’s instructions. Xylene and alcohol were used to dewaxing of the sample, and then deal with protease K. The sample was deal with denaturing solution in 78°C for 8 min and then alcohol was used for degeneration. The LINC00152 Probe was used to co-incubate with the sample in 37°C for 12–16 h. The optical microscope was used to observe the staining intensity and area.

### RNA Immunoprecipitation (RIP) and RNA Pull-Down

The RIP assays and RNA pull-down assay were conducted as previously shown in a study (18). Based on the manufacturer’s protocol, the RIP analysis was conducted using an EZ-Magna RIP Kit (17-701, Millipore). The quantitative RT-PCR was used to detect the purified, immunoprecipitated RNA and input genomic RNA. Biotin-labeled RNA was transcribed using the T7 RNA polymerase (Roche 10881775001) and Biotin RNA Labeling Mix (Roche 11685597910), mixed with recombinant DNase I (Roche 04716728001) and purified using an RNeasy Mini Kit (74904; Qiagen, Hilden, Germany). The NE-PER^®^ Nuclear and Cytoplasmic Extraction Reagents (78833; Pierce, Waltham, MA, USA) was used to extract the nuclear proteins were extracted from cells. The biotin-labeled were mixed with RNA cell nuclear extracts and washed streptavidin agarose beads (Sigma-Aldrich, St. Louis, MO, USA) and then added to each reaction. Five micrograms of anti-KLF5 antibody (ab237635, Abcam) were utilized to pull down RNA. The Flag-MS2bp-MS2bs system was used to perform the RNA pull-down assay. The bound proteins were analyzed by western blotting assay.

### Chromatin Immunoprecipitation-qPCR Assay

Based on the manufacturer’s instructions, a chromatin immunoprecipitation (ChIP) Kit (Millipore-Upstate) was used to immunoprecipitate DNA. Determine the number of the strip wells required and put these strips in the plate frame. Wash strip wells once with 150 μl of CP1. Add 100 μl of CP2 to each well and then add the antibodies: 1 μl of normal mouse IgG as the negative control, 1 μl of anti RNA polymerase II as the positive control, and 24 μg of each antibody of interest. Cover the strip wells with parafilm M and incubate at room temperature for 60–90 min. Meanwhile, prepare the cell extracts with 9 ml fresh culture medium containing 1% formaldehyde, and then incubate at room temperature for 10 min on an orbital shaker. Cell Lysis were proceeded by using glycine solution, CP3A and CP3B containing protease inhibitor cocktail to re-suspend the nuclear pellet. Shear DNA by sonication. Protein/DNA immunoprecipitation were proceeded by using CP4. Transfer 100 μl of diluted supernatant to each strip well. Cover the strip wells with parafilm M and incubate at room temperature for 60–90 min on an orbital shaker. Add 1 μl of proteinase K to each 40 μl of CP5 and mix. Add 40 μl of CP6 to the samples, mix, and recover the wells with strip caps and incubate at 65°C in a water bath for 90 min collection tube. Add 150 μl of CP7 to the samples and transfer mixed solution to the column vial. Add 10–20 μl of CP8 directly to the filter in the column and centrifuge at 12,000 rpm for 20 s to elute purified DNA. The purified DNA was measured *via* qPCR method (19).

### Luciferase Assay

Luciferase assay was performed as previously reported (19). By using the Lipofectamine 2000 transfection reagent (Thermo Fisher Scientific), wild-type LINC00152 promoter or mutant KLF5 promoter was transfected into MDA-MB-231 cells. The pRL-TK Renilla plasmid (Promega) was utilized as control. Subsequently, The Luciferase and Renilla signals was measured using a Dual-Luciferase Reporter Assay Kit (Promega).

### Western Blot Assay and Antibodies

The Western blot assay was performed as previously reported (20). The specific antibodies used were KLF5 (1:1,000, ab237635, Abcam), PTEN (1:1,000, ab76431, Abcam), β-catenin (1:1,000, ab237982, Abcam), BAP1 (ab199396, 1:500, Abcam), and GAPDH (1:1,000, ab245355, Abcam).

### Xenografts

Female BALB/c nude mice weighing about 15–18 g (SLAC, Shanghai, China) were housed under pathogen-free conditions. After being washed twice with serum-free medium, MDA-MB-231/MDA-MB-468 cells were reconstituted in serum-free Dulbecco’s Modified Eagle’s Medium and mixed with Matrigel (Becton-Dickinson) in a 1:1 ratio. After that, those mixed cells (2 × 10 (5)) were subcutaneously implanted into the right flank of each nude mouse. Permission was obtained from the Guidance of Institutional Animal Care and Use Committee of the Zhejiang Provincial People’s Hospital. All experimental procedures were conducted in according to the institutional animal regulations. The anesthetic used was 5% chloral hydrate, and the dose used was according to the weight of the mice (0.5 ml/kg). The animals used in this study were euthanized by CO_2_ inhalation in separate euthanasia cases using CO_2_ gas cylinders.

### Statistical Analysis

Statistical analyses were conducted using the GraphPad Prism software (GraphPad, version 5.0). The significance of data from patient’s specimen were determined by calculating Pearson’s correlation coefficient. The significance of the data between experimental groups *in vitro* and *in vivo* were tested using the Student’s test or Mann–Whitney U-test. P < 0.05 was considered statistically significant.

## Results

### LINC00152 Is Overexpressed in Breast Cancer and Related With Poor Prognosis

**Figure 1:**
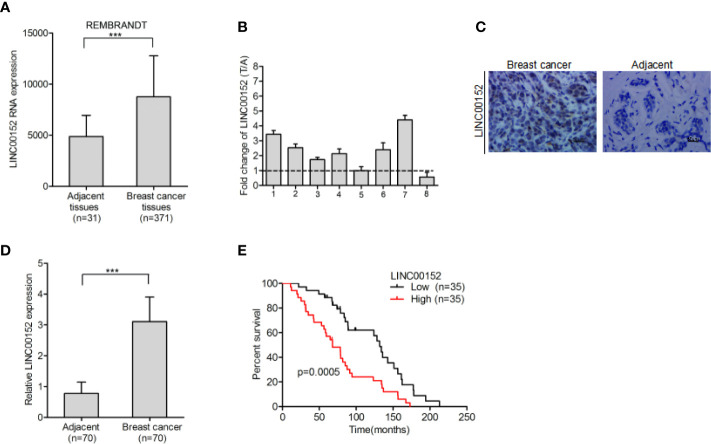
LINC00152 is overexpressed in breast cancer and indicate poor prognosis. **(A)** LINC00152 is overexpressed in breast cancer cells in comparison with the adjacent normal tissues in the REMBRANDT database. **(B)** LINC00152 expression was detected by qRT-PCR in eight pairs of freshly frozen human breast cancer tissues and adjacent normal tissues. **(C)** Representative images of LINC00152 expression in paraffin-embedded breast cancer and paired adjacent non-tumor specimens using RNAscope. Scale bars: 50 µm. **(D)** Quantification of LINC00152 expression in **(C)**. **(E)** Kaplan–Meier analysis of overall survival (OS) based on LINC00152 expression in all the 70 breast cancer patients. Error bars represent the SD of each value. ***P < 0.001. Data represent three independent experiments.

The REMBRANDT dataset (http://www.betastasis.com) was downloaded in order to examine the expression pattern of LINC00152 in breast cancer. We found that LINC00152 is dramatically upregulated in breast cancer tissues in comparison with adjacent normal tissues (**Figure 1A**). Additionally, LINC00152 was also upregulated in eight pairs of freshly frozen breast cancer tissues (**Figure 1B**). We used RNA *in situ* hybridization to detect LINC00152 expression in 70 pairs of paraffin-embedded breast cancer tissues and its adjacent normal tissues. We found that the expression of LINC00152 was significantly higher in tumor tissues than in adjacent normal tissues (**Figures 1C, D**), which echoed the REMBRANDT database result. We demonstrated that LINC00152 is abundant within both the nucleus and cytoplasm of breast cancer (**Figure 1C**). To evaluate the prognostic significance of LINC00152 expression in breast cancer, we used Kaplan–Meier analysis to evaluate the impact of LINC00152 expression on the survival rates of breast cancer patients. The data showed that the patients with relatively high LINC00152 expression (>median level) exhibited poorer prognosis than those with low LINC00152 expression (**Figure 1E**), suggesting that LINC00152 is overexpressed in breast cancer and is related with bad clinical outcome.

### Effects of LINC00152 on the Cell Proliferation and Tumorigenicity

**Figure 2:**
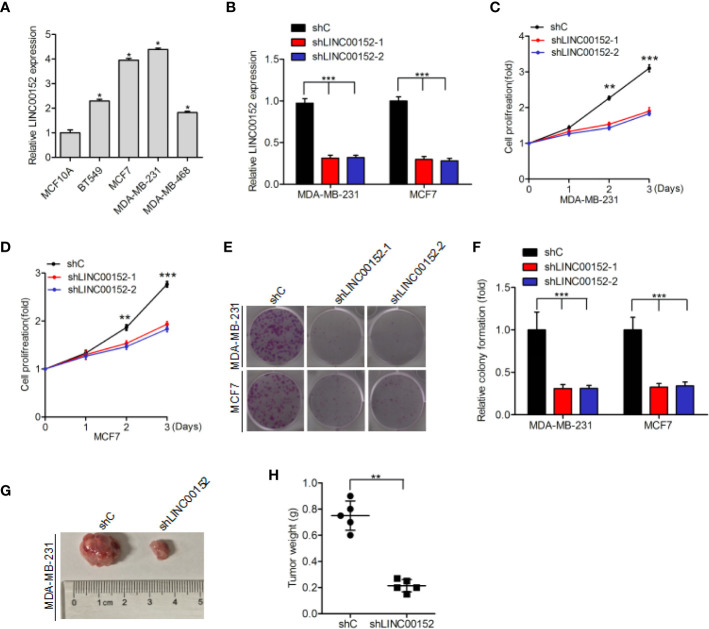
Impact of LINC00152 on the proliferation and tumorigenicity of breast cancer cells. **(A)** LINC00152 is upregulated in breast cancer cells (BT549, MCF7, MDA-MB-231, and MDA-MB-468) compared to the normal breast epithelium cells (MCF10A). **(B)** Knockdown of LINC00152 *via* two shRNAs (shLINC00152-1 and shLINC00152-2) or control shRNA (shC) in MCF7 and MDA-MB-231 cells. **(C–E)** Knockdown of LINC00152 inhibited cell proliferation **(C**, **D)** and colony formation **(E)**. **(F)** Quantification of colony formation in **(E)**. **(G)** Representative images of shC- or shLINC00152-infected MDA-MB-231 cells that were transduced when injected into nude mouse gland fat pads. Data were from three independent experiments with five mice per group. **(H)** Quantification of tumor weight in **(G)**. Error bars represent the SD of each value. *P < 0.05, **P < 0.01, ***P < 0.001. Data represent three independent experiments.

Quantitative real-time PCR assays indicated that LINC00152 expression was markedly upregulated in four breast cancer cell lines (BT549, MCF7, MDA-MB-231, and MDA-MB-468) compared with that in the normal breast epithelial cell line (MCF10A) (**Figure 2A**). We used shRNAs to deplete endogenous LINC00152 in MDA-MB-231 and MCF7 cell lines (**Figure 2B**), and found that LINC00152 knockdown dramatically suppress cellular proliferation (**Figures 2C, D**) and colony formation (**Figures 2E, F**). Moreover, LINC00152 knockdown (shLINC00152-1) also reduced breast cancer cell tumorigenicity *in vivo* (**Figures 2G, H**). In conclusion, our results indicate that LINC00152 significantly contributed to the growth and tumorigenicity of breast cancer.

### LINC00152 Interacts With KLF5 in Breast Cancer

**Figure 3:**
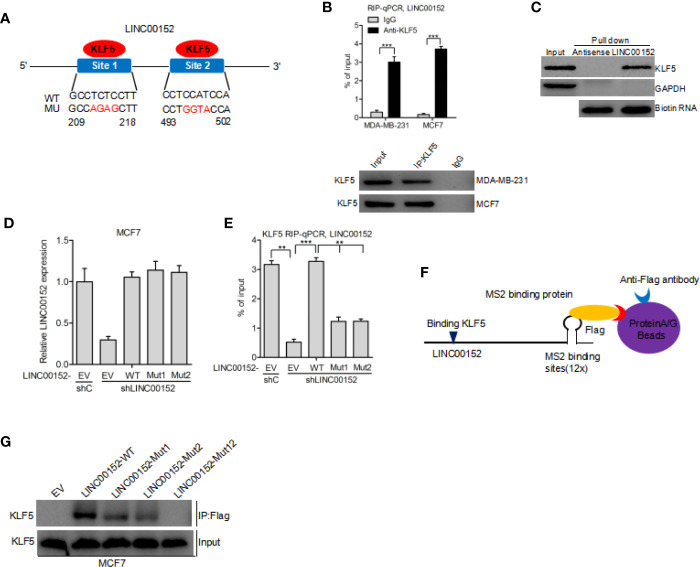
LINC00152 interacts with KLF5 in breast cancer. **(A)** Schematic diagram of putative KLF5 binding sites in LINC00152. **(B)** RIP-qPCR assay of the association of KLF5 with LINC00152 in MDA-MB-231 and MCF7 cells. **(C)** RNA pull-down assay shows that LINC00152 binds to KLF5. **(D)** Re-expression of shRNA-resistant LINC00152 wild type and KLF5-binding mutant types. **(E)** RIP-qPCR assay of effects of re-expression of shRNA-resistant LINC00152 wild type or mutant types on KLF5 binding. **(F**–**G)** Western blot detection of KLF5 after Flag-MS2bp-MS2bs-based pull-down assay (22). EV, empty vector. **P < 0.01, ***P < 0.001. Error bars represent SD. Data are representative of two or three independent experiments.

By using the Jaspar (http://jaspar.genereg.net/) database, we predicted that KLF5 may bind to LINC00152 at both 209 to 218 bp and 493 to 502 bp sites (Binding scores of the two regions are highest and their strands are +) (21) (**Figure 3A**). Hence, we performed RIP-qPCR analysis to determine whether KLF5 binds to LINC00152 in breast cancer cells. We assessed the enrichment of LINC00152 with the anti-KLF5 antibody in comparison with the control (**Figure 3B**). In addition, the RNA pull-down assay further demonstrated that LINC00152 binds with KLF5 protein (**Figure 3C**). To prove the hypothesis that LINC00152 binds to KLF5, we built LINC00152 vectors with mutations at the putative LINC00152-KLF5-binding site 1 (Mut1), site 2 (Mut2), and both sites (Mut12). We found that re-expression of shRNA-resistant LINC00152 wild-type (WT) rescued the combination of LINC00152 and KLF5, while re-expression of shRNA-resistant LINC00152-KLF5-binding Mut1 and Mut2 did not (**Figures 3D, E**). These results indicated that both of the two sites are essential for LINC00152-KLF5 binding. In order to further prove that LINC00152 binds to KLF5, the RNA pull-down assay was performed by using the Flag-MS2bp-MS2bs system (22), wherein the FLAG-tagged MS2-binding protein (MS2BP) could directly binds to RNA that contains MS2-binding sequences (**Figure 3F**). As shown in **Figure 3G**, the result indicated that KLF5 could bind to the wild-type of LINC00152 but not to the mutant in MCF7 cells. These results convincingly indicated that KLF5 binds to LINC00152 in breast cancer cells.

### LINC00152 Regulates the Stability of KLF5 Protein

**Figure 4:**
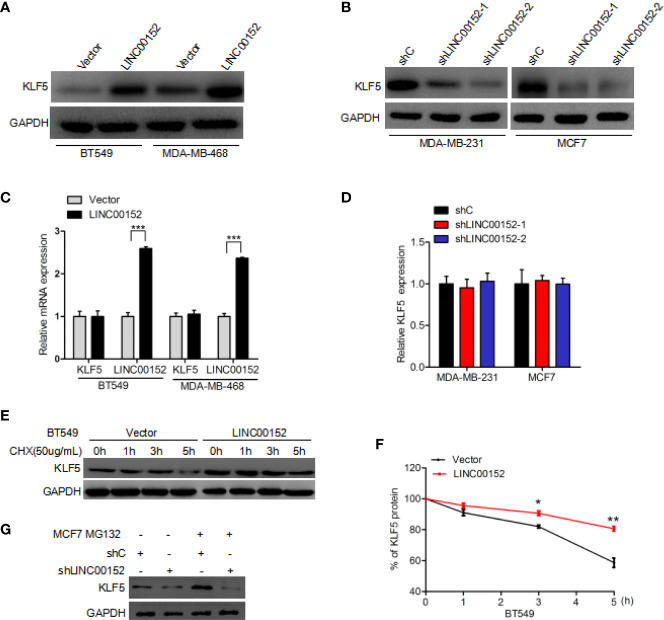
LINC00152 regulates KLF5 protein stability. **(A)** Overexpression of LINC00152 increases the expression level of KLF5 protein. **(B)** Knockdown of LINC00152 suppresses KLF5 protein expression. **(C)** qRT-PCR analysis of effect of LINC00152 overexpression on KLF5 mRNA expression. **(D)** qRT-PCR analysis of effect of LINC00152 depletion on KLF5 mRNA expression. **(E)** Effect of LINC00152 overexpression on KLF5 degradation. Cycloheximide (20 mg/ml) was added to treat cells for the indicated time. **(F)** Quantification of KLF5 protein expression in **(E)**. **(G)** Effect of LINC00152 depletion on KLF5 degradation. MG132 was added to treat cells for the indicated time. *P < 0.05, **P < 0.001. Error bars represent SD. Data are representative of three independent experiments.

To determine the exact role of KLF5 in LINC00152-mediated breast cancer growth, we detected the expression of KLF5 at mRNA and protein level in LINC00152 knockdown breast cancer cells. In comparison with the control, overexpression of LINC00152 dramatically increased the expression level of KLF5 protein in BT549 and MDA-MB-468 cells (**Figure 4A**). Moreover, knockdown of LINC00152 could dramatically decrease the expression level of KLF5 protein both in MCF7 and MDA-MB-231 cells (**Figure 4B**). However, the mRNA level of KLF5 was not affected by either overexpression or depletion of LINC00152 (**Figures 4C, D**). These results, taken together, demonstrated that LINC00152 might regulate the stability of KLF5 protein. In order to validate the hypothesis, an inhibitor of *de novo* protein synthesis, cycloheximide, was utilized to treat BT549 cells at the indicated time points (**Figure 4E**). As a result, the KLF5 protein levels was dramatically decreased after cycloheximide treatment at the 5-h mark in BT549 cell lines compared with the untreated controls (**Figures 4E, F**). Moreover, overexpression of LINC00152 markedly decreased KLF5 degradation (**Figures 4E, F**). Furthermore, MCF7 cells were also treated with MG132 (23, 24), an inhibitor of protein degradation. In comparison with the controls, knockdown of LINC00152 dramatically increased the KLF5 degradation in MCF7 cell lines (**Figure 4G**). The deubiquitinase (DUB) BAP1 had been reported to regulate KLF5 stability in breast cancer cells (19, 25), so we hypothesize that LINC00152 mediates KLF5 depending on the binding of BAP1 and KLF5. As shown in **Supplementary Figure 1**, LINC00152 knockdown inhibits BAP1 binding with KLF5. These data show that LINC0015 can regulate the stability of KLF5 protein in breast cancer cells.

### KLF5 Regulates LINC00152 Expression *via* Binding to the LINC00152 Promoter

**Figure 5:**
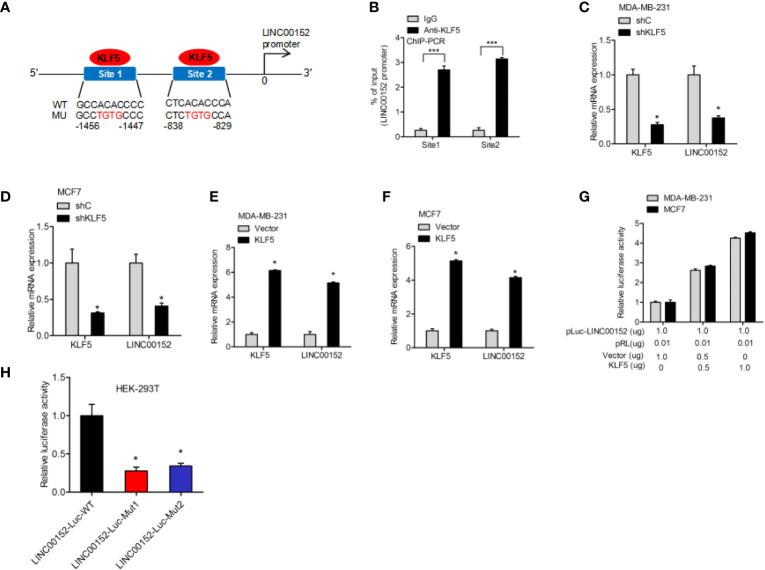
>KLF5 regulates LINC00152 expression through LINC00152 promoter binding. **(A)** Schematic diagram of putative KLF5-binding sites in LINC00152 promoter. **(B)** ChIP-qPCR assay of KLF5 binding to LINC00152 promoter. **(C, D)** qRT-PCR analysis of effect of KLF5 depletion on LINC00152 expression in MDA-MB-231 and MCF7 cells. **(E, F)** qRT-PCR analysis of effect of KLF5 overexpression on LINC00152 expression in MDA-MB-231 and MCF7 cells. **(G)** Luciferase assay of KLF5 regulation on the LINC00152 promoter activity in MDA-MB-231 and MCF7 cells. **(H)** Luciferase assay of KLF5 site regulation on the LINC00152 promoter activity in HEK-293T cells. *P < 0.05. ***P < 0.001. Error bars represent SD. Data are representative of three independent experiments.

To further explore the underlying regulatory mechanism, we predicted the potential transcription factor-binding sites of KLF5 in LINC00152 by using the Jaspar database. It was predicted that KLF5 could bind to the LINC00152 promoter at both −1456 to −1447 bp and −838 to −829 bp sites (**Figure 5A**). Therefore, the ChIP assay was performed to determine whether KLF5 binds to the promoter of LINC00152 at those two possible sites (**Figure 5B**). KLF5 knockdown inhibited LINC00152 expression (**Figures 5C, D**), while the overexpression of KLF5 promoted LINC00152 expression in MCF7 and MDA-MB-231 cells (**Figures 5E, F**). The dual-luciferase reporter assays further suggested that KLF5 overexpression stimulated the promoter activity of LINC00152 in MCF7 cells (**Figure 5G**). To further validate the hypothesis, we built LINC00152 vectors with mutation at the potential KLF5-LINC00152 promoter-binding site 1 (Mut1) and site 2 (Mut2). Compared with the wild-type LINC00152 promoter, both of the putative mutations dramatically suppress the transcriptional activity of the LINC00152 promoter, which convincingly prove that both of these two sites are essential for KLF5-LINC00152 promoter binding (**Figure 5H**).

### LINC00152 Promotes Breast Cancer Cell Proliferation *via* KLF5

**Figure 6:**
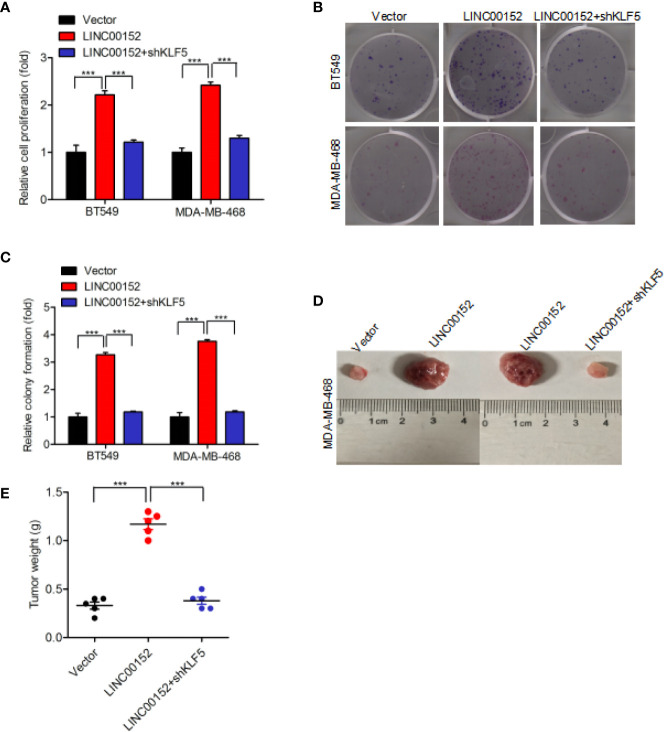
LINC00152 promotes cell proliferation *via* KLF5 in breast cancer. **(A, B)** Knockdown of KLF5 restores LINC00152 overexpression-promoted cell proliferation **(A)** and colony formation **(B)** in BT549 and MDA-MB-486 cells. **(C)** Quantification of colony formation in **(B)**. **(D)** Representative images of LINC00152 overexpression or LINC00152 overexpression combined with KLF5 knockdown groups-infected MDA-MB-468 cells that were transduced when injected into nude mouse gland fat pads. Data were from three independent experiments with five mice per group. **(E)** Quantification of tumor weight in **(D)**. ***P < 0.001. Error bars represent SD. Data are representative of three independent experiments.

Our result also found that overexpression of LINC00152 significantly facilitated the proliferation of breast cancer cell, whereas knockdown of KLF5 did not induce cancer cell proliferation (**Figure 6A**). Subsequent assays revealed that KLF5 knockdown restored the overexpression of LINC00152-mediated enhancement of colony formation (**Figures 6B, C**). Moreover, KLF5 knockdown also reduced the enhancement of breast cancer cell tumorigenicity through overexpression of LINC00152 (**Figures 6D, E**). In conclusion, these results demonstrated that LINC00152 facilitate the breast cancer cell proliferation and tumorigenicity through KLF5.

### Effect of the LINC00152-KLF5 Loop on PTEN and β-Catenin Expression in Breast Cancer

Previously published studies reported that LINC00152 could directly inhibit PTEN expression (5), and KLF5 can directly promote β-catenin expression in breast cancer (19). To explore the correlation between the LINC00152-KLF5 loop and those proteins, we tried to determine whether LINC00152 or KLF5 affected the expression of PTEN or β-catenin in breast cancer cells. In consequence, we found that the overexpression of LINC00152 or KLF5 dramatically downregulated the expression level of PTEN and upregulated β-catenin protein in BT549 cell line. Additionally, the result also indicated that the knockdown of LINC00152 can revert the effects of KLF5 on PTEN and β-catenin protein expression and *vice versa* (**Supplementary Figure 2**). There is a positive loop effect of LINC00152-KLF5 on breast cancer cell proliferation (**Supplementary Figure 3**). Moreover, based on quantification of the mRNA expression, LINC00152 was revealed to markedly correlate with KLF5 using Spear-man’s rank correlation analysis (**Supplementary Figure 4**). Taken together, the data suggests that LINC00152 regulates the downstream proteins of KLF5 and *vice versa*.

## Discussion

Our result demonstrated that lncRNA LINC00152 plays a vital role in breast cancer tumor growth and proliferation in a KLF5-mediated manner. LINC00152 is dramatically overexpressed in clinical breast cancer tissues in comparison with paired normal tissues. Breast cancer patients with relatively high LINC00152-expressing had a poorer clinical outcome. Additionally, LINC00152 directly bound to KLF5 and stabilized KLF5 by regulating protein degradation, resulting in enhanced tumorigenesis. KLF5 also directly bound to the LINC00152 promoter and thus activate the transcription of LINC00152. LINC00152 is a KLF5-responsive lncRNA.

Accumulating evidences suggested that abnormal expression of lncRNA played a critical role in the development of breast cancer (26, 27). Recently, LINC00152 was reported to be amplified in several cancers (7–10), including breast cancer (4–6). For instance, LINC00152 has been shown to promote tumorigenesis in triple-negative breast cancer by regulating DNMTs or PTEN (5, 6). In current study, we found that LINC00152 is markedly upregulated in breast cancer, and a relatively higher expression level of LINC00152 was significantly associated with poor survival in breast cancer patients. Knockdown of LINC00152 suppress the colony formation, cell proliferation, and tumor growth of breast cancer.

Importantly, we proved a positive feedback loop between LINC00152 and KLF5. Here, we revealed that LINC00152 binds to KLF5 and regulates KLF5 expression. Using Jaspar, we found that LINC00152 binds to KLF5 at two sites (21). It would be of interest to determine how LINC00152 regulates KLF5 expression. As a transcription factor, KLF5 belongs to a family of zinc-finger-containing genes which plays a vital role in regulating a wide range of genes. It was reported that KLF5 could bind to promoters of downstream effector genes and stimulate the transcriptional activities, thereby affecting diverse cellular functions (28, 29). KLF5 was also reported to play a critical role in breast carcinogenesis (28). Our previous study revealed that lncRNA PVT1 contributes the development of triple-negative breast cancer *via* KLF5 (19). Through western blotting and qRT-PCR, we found that LINC00152 regulates KLF5 protein expression without affecting KLF5 RNA expression. Knockdown of LINC00152 inhibited KLF5 protein *via* enhanced KLF5 degradation. In contrast, LINC00152 overexpression promoted KLF5 protein *via* inhibition of KLF5 degradation. Therefore, our findings demonstrate that LINC00152 directly binds to KLF5 protein and increases KLF5 protein post-translationally.

The correlation between KLF5 and LINC00152 in breast cancer was largely unclear. Our study revealed that KLF5 bound to the promoter of LINC00152 and regulated its transcription. By using Jaspar, we firstly identified two binding sites of KLF5 on the LINC00152 promoter in breast cancer (21). KLF5 could directly bind to these two sites and thus activate LINC00152 transcription. This result was similar to findings obtained in our previously reported work (16). The ChIP assay demonstrated that KLF5 binds to the LINC00152 promoter at site 1 and site 2. Additionally, by using luciferase analyses, we reported that KLF5 overexpression promoted LINC00152 expression *via* enhanced LINC00152 promoter activity. In contrast, knockdown of KLF5 inhibited LINC00152 expression by inhibiting LINC00152 promoter activity.

It has been shown that lncRNAs could function in post-transcriptional processes (30, 31). Moreover, lncRNAs was also demonstrated to affect its own expression *via* a positive feedback loop through functioning as a decoy (32). A recent study demonstrated a positive feedback loop between lncRNA-PVT1 and FOXM1 in gastric cancer (24). Similarly, our study also exhibited a positive feedback loop between LINC00152 and KLF5 in breast cancer. Therefore, a reciprocal regulatory mechanism might be frequently between lncRNAs and transcriptional factors, which exerted their oncogenic function mutually to facilitate cancer progression.

Additionally, knockdown of KLF5 restored LINC00152 overexpression-induced cell proliferation, colony formation, and tumorigenicity. Previously published studies reported that LINC00152 could directly inhibit PTEN (5), and KLF5 could directly promote β-catenin (19). In current study, we demonstrated that both LINC00152 and KLF5 affected the protein levels of PTEN and β-catenin. This suggests that LINC00152 and KLF5 can regulate the downstream genes of each other.

In summary, our study demonstrated LINC00152 as an oncogenic lncRNA in breast cancer. We have also unveiled a novel mechanism by which LINC00152 stabilizes KLF5 *via* post-translational regulation. Meanwhile, KLF5 directly bound to the promoter of LINC00152 and thus activate its transcription, which ultimately enhances tumorigenesis in breast cancer. These findings may aid in the identification of novel biomarkers for LINC00152 and may provide a theoretical basis for the LINC00152-KLF5 loop-mediated treatment of breast cancer in the future.

## Data Availability Statement

The raw data supporting the conclusions of this article will be made available by the authors, without undue reservation.

## Ethics Statement

The studies involving human participants were reviewed and approved by the Ethics Committee of Zhejiang Provincial People’s Hospital. Written informed consent for participation was not required for this study in accordance with the national legislation and the institutional requirements. The animal study was reviewed and approved by the Guidance of Institutional Animal Care and Use Committee of the Zhejiang Provincial People’s Hospital.

## Author Contributions

QL, MJ, MZ, LZ, and GZ: performed *in vivo* experiments and animal experiments and contributed to the study design. SX and GZ: performed WB analysis. YZ, HZ, XW, ZL, and XL: contributed to the data analysis and writing of the manuscript. JT and HZ: conceived and supervised the study, provided funding, and wrote the manuscript. All authors contributed to the article and approved the submitted version.

## Funding

This study was supported in part by grants from National Natural Science Foundation of China (Grant numbers: 81802626, and 81972854 to LZ), National Natural Science Foundation of China (Grant number: 82003215 to JT), Zhejiang Provincial Nature Science Foundation of China (Grant number: LQ20H160063 to JT), Zhejiang Provincial People’s Hospital Scientific Research Returned Foundation for the Excellent Youth (Grant number: ZRY2018B002 to JT), National Natural Science Foundation of China (Grant number: 82003236 to HZ), Zhejiang Provincial People’s Hospital Scientific Research Returned Foundation for the Excellent Youth (Grant number: ZRY2018C009 to HZ), Shanghai Jiao Tong University Medical Engineering Cross Fund (Grant number: YG2017QN49 to LZ), Zhejiang Provincial Nature Science Foundation of China (Grant number: LQ20H160030 to GZ), and Nurturing Fund of Renji Hospital (Grant number: PYZY16-018 to LZ).

## Conflict of Interest

The authors declare that the research was conducted in the absence of any commercial or financial relationships that could be construed as a potential conflict of interest.
