# Supplementary figures and images for: A Positive Feedback Loop of Long Noncoding RNA LINC00152 and KLF5 Facilitates Breast Cancer Growth
Source: Front Oncol. 2021 Mar 26;11:619915. doi: 10.3389/fonc.2021.619915 (PMC8032978; doi:10.3389/fonc.2021.619915)

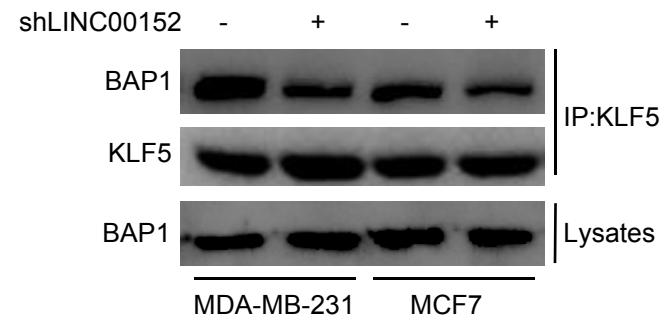

**Supplementary Figure 1** Effects of LINC00152 knockdown on KLF5-BAP1 binding .

Supplement: Supplementary file 1 [file DataSheet_1.pdf]
