# Supplementary material for: A Positive Feedback Loop of Long Noncoding RNA LINC00152 and KLF5 Facilitates Breast Cancer Growth
Source: Front Oncol. 2021 Mar 26;11:619915. doi: 10.3389/fonc.2021.619915 (PMC8032978; doi:10.3389/fonc.2021.619915)
Supplement: Supplementary file 2 [file DataSheet_2.pdf]

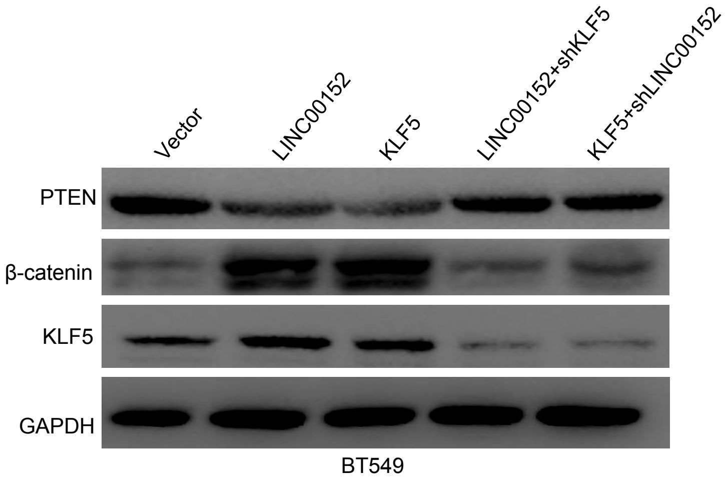

Supplementary Figure 2 Western blotting detection reveals LINC00152 or KLF5 knockdown rescued KLF5 or LINC00152 overexpression-altered PTEN, β-catenin or KLF5 protein and expression.
