# Supplementary material for: A Positive Feedback Loop of Long Noncoding RNA LINC00152 and KLF5 Facilitates Breast Cancer Growth
Source: Front Oncol. 2021 Mar 26;11:619915. doi: 10.3389/fonc.2021.619915 (PMC8032978; doi:10.3389/fonc.2021.619915)
Supplement: Supplementary file 3 [file DataSheet_3.pdf]

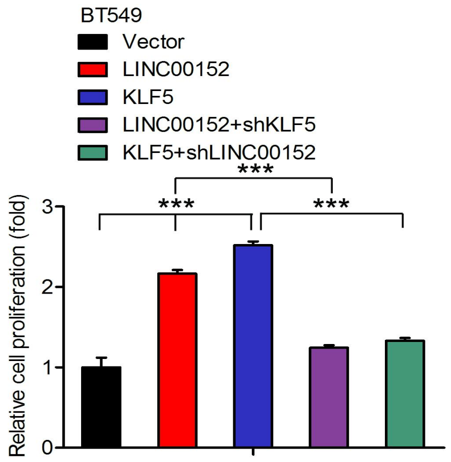

Supplementary Figure 3 LINC00152 or KLF5 knockdown rescued KLF5 or LINC00152 overexpression-inhibited cell proliferation. Error bars represent the SD of each value. ***P< 0.001.Data represent three independent experiments.
