# Supplementary material for: A Positive Feedback Loop of Long Noncoding RNA LINC00152 and KLF5 Facilitates Breast Cancer Growth
Source: Front Oncol. 2021 Mar 26;11:619915. doi: 10.3389/fonc.2021.619915 (PMC8032978; doi:10.3389/fonc.2021.619915)
Supplement: Supplementary file 4 [file DataSheet_4.pdf]

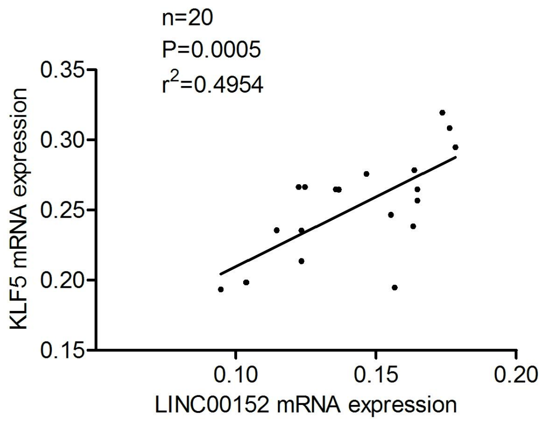

Supplementary Figure 4 Correlation of expression of LINC00152 with KLF5 in 20 snap-frozen clinical breast cancer tumor specimens.
