# Supplementary material for: A Positive Feedback Loop of Long Noncoding RNA LINC00152 and KLF5 Facilitates Breast Cancer Growth
Source: Front Oncol. 2021 Mar 26;11:619915. doi: 10.3389/fonc.2021.619915 (PMC8032978; doi:10.3389/fonc.2021.619915)
Supplement: Supplementary file 5 [file Table_1.docx]

**Supplementary Table 1** Primers for qRT-PCR assays

| Primer pairs | Sequence |
| --- | --- |
| qRT-PCR,GAPDH | 5’-GGAGCGAGATCCCTCCAAAAT-3’ and  5’-GGCTGTTGTCATACTTCTCATGG-3’ |
| qRT-PCR,LINC00152 | 5’-AAAATCACGACTCAGCCCCC-3’ and  5’-AATGGGAAACCGACCAGACC -3’ |
| qRT-PCR,KLF5 | 5’-CCTGGTCCAGACAAGATGTGA-3’ and  5’-GAACTGGTCTACGACTGAGGC-3’ |
